# Diabetes Mellitus, Cognitive Impairment, and Traditional Chinese Medicine

**DOI:** 10.1155/2015/810439

**Published:** 2015-04-28

**Authors:** S. W. Seto, G. Y. Yang, H. Kiat, A. Bensoussan, Y. W. Kwan, D. Chang

**Affiliations:** ^1^National Institute of Complementary Medicine, University of Western Sydney, Campbelltown, NSW 2560, Australia; ^2^Faculty of Medicine, University of New South Wales, Kensington, NSW 2052, Australia; ^3^School of Medicine, University of Western Sydney, Locked Bag 1797, Penrith, NSW 2751, Australia; ^4^Faculty of Medicine and Health Sciences, Macquarie University, NSW 2109, Australia; ^5^School of Biomedical Sciences, The Chinese University of Hong Kong, Shatin, Hong Kong

## Abstract

Diabetes mellitus (DM) is a metabolic disorder affecting a large number of people worldwide. Numerous studies have demonstrated that DM can cause damage to multiple systems, leading to complications such as heart disease, cancer, and cerebrovascular disorders. Numerous epidemiological studies have shown that DM is closely associated with dementia and cognition dysfunction, with recent research focusing on the role of DM-mediated cerebrovascular damage in dementia. Despite the therapeutic benefits of antidiabetic agents for the treatment of DM-mediated cognitive dysfunction, most of these pharmaceutical agents are associated with various undesirable side-effects and their long-term benefits are therefore in doubt. Early evidence exists to support the use of traditional Chinese medicine (TCM) interventions, which tend to have minimal toxicity and side-effects. More importantly, these TCM interventions appear to offer significant effects in reducing DM-related complications beyond blood glucose control. However, more research is needed to further validate these claims and to explore their relevant mechanisms of action. The aims of this paper are (1) to provide an updated overview on the association between DM and cognitive dysfunction and (2) to review the scientific evidence underpinning the use of TCM interventions for the treatment and prevention of DM-induced cognitive dysfunction and dementia.

## 1. Introduction

Diabetes mellitus (DM) is a metabolic disorder characterised by an increase in plasma glucose level due to insulin deficiency and/or resistance that can lead to damage to multiple organs. Currently, approximate 347 million people are suffering from DM worldwide and the number will continue to increase. There are two types of DM: type 1 diabetes mellitus (T1DM) and type 2 diabetes mellitus (T2DM). T1DM is caused by the destruction of the pancreatic *β*-cells due to an autoimmune reaction, leading to absolute insulin deficiency, whilst T2DM is characterised by insulin resistance, where the body fails to produce an appropriate physiological response to circulating insulin. T2DM accounts for approximately 90% of all DM cases with the prevalence increasing with age. Tremendous effort has been invested to understand the complications of DM and its impact on vision loss, neuropathy, and cardiovascular diseases; however, DM-induced cognitive dysfunction is seldom addressed and is not as well understood.

Since life expectancy has been markedly prolonged with advances in medicine, it has been suggested that the incidence of T2DM and dementia would increase as the population ages [1, 2]. Indeed, several epidemiological studies have shown that people with T2DM have a significantly higher risk of developing cognitive impairments and dementia when compared to those with normal blood glucose levels [3–5]. A recent population-based longitudinal study has shown that the relative risk of Alzheimer's diseases (AD) and vascular dementia (VaD) in the DM population was 1.46 (95% CI: 1.20–1.77) and 2.5 (95% CI: 2.1–3.0), respectively, when compared to people without DM [6]. Moreover, DM has been suggested to be an individual risk factor for dementia [7, 8], independent of other established risk factors, such as hypertension and atherosclerosis [8, 9].

The brain pathology underlying cognitive dysfunction is heterogeneous and is highly complicated. Traditionally, AD is considered as the major diagnosis of dementia [10]; however numerous clinical-pathological studies have suggested a significant contribution of cerebrovascular diseases to cognitive decline [11, 12]. Although the exact pathophysiology of DM-mediated dementia has not been fully elucidated, existing evidence has shown that both cerebrovascular changes and neurodegeneration are implicated in the development and progression of DM-mediated cognitive dysfunction [5]. To date, there are no DM-specific treatments to prevent or ameliorate cognitive dysfunction. Nevertheless, numerous reports have highlighted the therapeutic potential of antidiabetic therapies in the treatment and prevention of cognitive dysfunction [13–15]. In the largest randomized controlled trial to date, the ACCORD-MIND study, it was shown that the decline in total brain volume was significantly reduced in the intensive glycemic control group, compared to the standard glycemic control group. Although the cognitive outcomes were not different, the effect of glycemic control in preserving cerebral structure cannot be denied [16]. Findings from these studies have clearly indicated that treatments targeting DM could be a novel strategy to prevent dementia development and potentially to slow down the progression of cognitive dysfunction.

Through the advances in pharmacological therapy, many oral antidiabetic agents have become available. Interestingly, oral antidiabetic drugs such as thiazolidinedione and metformin have been shown to have beneficial effects to slow the progression of dementia in both clinical and animal studies [17, 18]. However, many of these pharmaceutical agents are associated with various undesirable side-effects, such as weight gain, fluid retention, and increased risk for heart failure, limiting their compliance and utility in clinical practice. Traditional Chinese medicine (TCM), including Chinese herbal medicines (CHM) and acupuncture, has been used for thousands of years for the management of disease, maintenance of health, and prolongation of life expectancy. Accumulated evidence suggests that many CHMs and their active ingredients possess hypoglycemic properties and that some TCM interventions have beneficial effects in the treatment and prevention of DM and its complications, with minimal toxicity and fewer adverse reactions [19]. In addition, existing evidence has demonstrated the therapeutic potential of TCMs in DM-mediated cognitive dysfunction [20]. In this paper, we present a comprehensive review of current understanding of DM-mediated dementia and the scientific research on the use of TCMs for the management of cognitive dysfunction in DM.

## 2. Association of Diabetes and Cognitive Dysfunction and/or Dementia

Poor glycemic control has been associated with progression of cognitive dysfunction [21]. An increasing number of studies have reported an acceleration of cognitive decline in patients with DM, independent of common cardiovascular risk factors [8, 22]. To date, DM is recognised as an independent risk factor for the development of cognitive dysfunction. In a meta-analysis based on twenty-five studies, it was estimated that T2DM patients have 1.5-fold greater risk of cognitive dysfunction and 1.6-fold increased risk of dementia, when compared to people without diabetes [23]. Similarly, a recent report has shown a 1.5-fold higher risk of AD in people with diabetes than those without diabetes [6]. Most reports so far have suggested an increased risk of global cognitive dysfunction in diabetes [6, 23, 24], while some reports showed more selective cognitive impairment, mainly affecting learning, mental speed, and visuospatial process [25–27]. It is important to point out that the discrepancy between these studies may simply be due to the variation of neurocognitive testing, such as age, education, sex, history of other illnesses, and the duration/severity of diabetes [28, 29].

Many complications of diabetes, such as retinopathy, lower limb ulcers, and atherosclerosis, usually take years to develop before becoming clinically apparent. However, cognitive function decrement has been observed in the early stage of T2DM [30]. In children with T1DM, deficits in cognitive development, including vocabulary, block design, general intelligence, speed of processing, and learning, have been observed as early as 2 years after the onset of T1DM [31]. These findings suggested that deficits in regulation of blood sugar level, even at an early stage, would have detrimental effects on cognitive function. Interestingly, recent studies have demonstrated that elevated blood glucose levels may be a risk factor for impaired cognitive function leading to dementia, even among people without diabetes [32, 33] highlighting the relationship between high blood glucose level and dementia outcome.

There is ample evidence from neuropsychological studies reporting that people who have DM also suffer from mild cognitive impairment (MCI). Longitudinal studies have shown that approximate 55% of patients with MCI developed probable Alzheimer's dementia over 3 years [34, 35] and the progression rate reached 100% after 9.5 years [36]. It has been suggested that DM patients have 50% higher chance of developing Alzheimer's disease than those without DM [6]. A longitudinal study has also shown a relationship between diabetes and incidence of MCI [37]. Progression of MCI to dementia has been shown to be markedly accelerated by diabetes in elderly subjects who were either cognitively intact or diagnosed with MCI at baseline [38]. Brain imaging studies have provided direct evidence to support DM-mediated MCI and dementia [39, 40]. Resting-state functional magnetic resonance imaging (rs-fMRI) studies have revealed abnormalities in amplitude of low-frequency fluctuations (ALFF) in T2DM patients in multiple brain regions. These present as decreased ALFF in the bilateral middle temporal gyrus and left fusiform gyrus and increased ALFF in bilateral cerebellum posterior lobe and right cerebellum culmen [41]. Moreover, recent studies have shown that alternation of ALFF and reduced connectivity of the hippocampus are associated with the presence of diabetic vascular disease and poor cognitive performance in T2DM patients [42, 43]. Although the mechanism between MCI and the increased risk of dementia under DM is not fully understood, it has been suggested that the DM-mediated MCI and dementia are not likely to form a continuum, given the difference in etiologies and risk factors between MCI and dementia [44, 45].

People suffering from DM over a long period have been shown to express an elevated level of dementia [46, 47]. There are an extensive number of studies examining the effect of DM on cognitive functions in elder population [48–50]. It has been shown that the prevalence of dementia in T2DM patients increased over age, from 2.4% in the age group of 65–76 and 5% in 76–85 to 8.3% for patients over 85 years of age [51]. Several studies have also reported an increased incidence of dementia in individuals who were diagnosed with DM in midlife after an extended follow-up of 25–35 years [7]. However, the exact effect of midlife against late-life DM onset on cognitive impairment and dementia remains to be clarified.

## 3. Pathophysiology of Cognitive Dysfunction and/or Dementia in Diabetes

**Figure 1:**
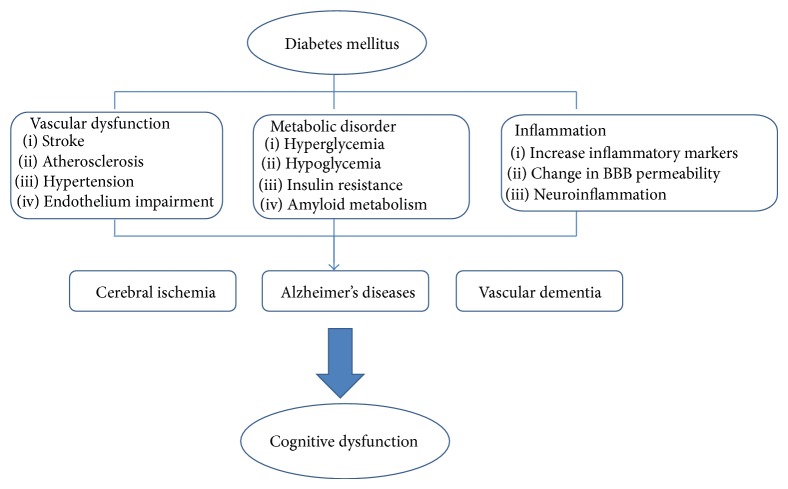
Mechanism of DM-mediated cognitive dysfunction.

The mechanisms underlying the development of cognitive dysfunction in diabetes have not been fully elucidated. Many hypotheses have been suggested based on the pathophysiological mechanisms through which diabetes might affect the initiation and progression of the pathology of dementia [52]. These proposed mechanisms include various diabetic-specific factors or signalling pathways that may influence cognitive functioning, such as hyperglycemia, insulin deficiency, microvascular complications, and inflammation. In this section, we will highlight some of the risk factors and possible mechanisms related to cognitive dysfunction in diabetes (Figure 1).

### 3.1. Vascular Dysfunction

Vascular complications, including atherosclerosis, hypertension, stroke, and vascular comorbidity, are closely associated with DM. Recent studies have reported that vascular complications are likely to be an important determinant of cognitive dysfunction and dementia [11]. Increased cerebral infarcts and reduction of amyloid-beta load were observed in older DM patients compared to nondiabetics [53]. A meta-analysis of longitudinal studies suggested that there is a stronger association of vascular-related cognitive impairment than AD with DM patients [6]. Interestingly, less Alzheimer's-like pathology has been observed, but more ischemic lesions in T2DM patients with a clinical diagnosis of dementia have been observed [54]. Indeed, an increasing number of studies are suggesting that the reduction of cerebral perfusion plays a significant role in the development of AD [55], supporting the hypothesis that cerebrovascular pathology such as stroke predisposes cognitive decline and dementia development. The detrimental effects of DM on cognitive function in vascular dementia have been demonstrated in a recent preclinical study. Kwon et al. showed that exacerbated cognitive functions caused by diabetes were mediated via augmentation of neuronal cell death in the hippocampus through CREB/BDNF signalling pathway in an animal model of vascular dementia [56].

Hypertension has been shown to be a significant risk factor for poor cognitive performance in both T1DM and T2DM patients [57, 58]. In addition to hypertension, atherogenic dyslipidemia is another common vascular risk factor in DM [59]. Dyslipidemia contributes to atherosclerosis development [60] and has been found to increase risk of dementia in diabetes [61]. Moreover, reduced cerebral blood flow [62], upregulation of inflammatory cytokines [63, 64], endothelial dysfunction [65], and abnormalities in cerebral capillaries [66] have been demonstrated in patients with diabetes. Changes in cerebral vasculature by these factors are closely associated with stroke and brain damage, including brain infarct and white matter lesions [67], contributing to cognition deterioration in diabetes. Given the complex pathophysiology of vascular complications in diabetes, more research is required to explore the exact mechanisms around how these DM-related vascular risks contribute to cognitive decline and dementia. However, majority of current findings have confirmed the association of vascular risk factors and cognitive decrements in diabetes and support the belief that predominant cerebrovascular pathology in diabetes could aggravate cognitive functioning.

### 3.2. Metabolic Abnormalities

Blood glucose levels are regulated by the endocrine system involving multiple organs and signalling molecules and pathways. Upset of this precisely regulated process could lead to imbalance of blood glucose level, resulting in organ damage. Although the exact mechanisms behind the association between DM and cognitive impairment or dementia are unclear, studies have shown that it is a multifactorial process where metabolic condition plays a significant role.

#### 3.2.1. Hyperglycemia

Chronic high blood glucose levels have been shown to have negative effects on cognitive functions and brain structure [68]. Hyperglycemia is a characteristic in both T1DM and T2DM. Numerous studies have demonstrated a close relationship between glucose intolerance and cognitive decrements and dementia [24, 69, 70]. It has been shown that people with poor glycemic control, with glycosylated hemoglobin (HbA1c) higher than 7.0%, have a 4-fold higher risk of developing cognitive impairment [71]. Similarly, an inverse association of HbA1c and cognitive function such as working memory, learning, and executive functioning has been observed in T2DM patients [72]. The results of these studies highlight the contribution of poor glycemic control in cognitive function deterioration process. Multiple toxic effects of hyperglycemia on the brain, such as formation of advanced glycated end products (AGEs), generation of reactive oxygen species (ROS), and activation of polyol, diacylglycerol, and hexosamine pathways, have been suggested.

It has been shown that hyperglycemia leads to enhanced formation of AGEs [73]. AGEs have been shown to contribute to microvascular complications, accelerated amyloid-beta deposition and senile plaque formation [9, 71]. A preclinical study has demonstrated that increased cerebral AGEs expression is associated with cognitive dysfunction in diabetic mice [74]. Similarly, increased AGEs levels have been observed in AD patients with T2DM, when compared to nondiabetic AD patients [75]. Moreover, AGEs lead to ROS generation via activation of a RAGE cell surface receptor for AGEs, which in turns leads to neuronal injury [76, 77].

It is well established that oxidative stress is implicated in both the onset and progression of diabetes and its complications. It has been shown that cognitive deficit caused by hyperglycemia in diabetic rat is associated with an increase in ROS levels and reduction of antioxidant levels [78, 79]. In addition, increased ROS generation has been shown to activate various cellular signalling pathways, such as the polypol pathway, protein kinase C activation, and increase of glucose shunting via the hexosamine pathway, all of which are related to neuronal injury and cerebral damage [80]. Interestingly, it was shown that administration of antioxidants could reverse the cognitive dysfunction in the diabetic rats [78, 79], suggesting a potential therapeutic target for DM-mediated cognitive impairment.

#### 3.2.2. Hypoglycemia

Sufficient glucose supply is vital for normal brain function and it is well established that hypoglycemia has detrimental effects on the brain [81, 82]. Repeated hypoglycemic episodes are a common side-effect in patients who receive intensive insulin therapy for diabetes [83]. In animal studies, it has been shown that exposure to low blood glucose levels can cause cerebral energy failure, neuronal necrosis, and brain damage leading to a flat electroencephalograph and cognition dysfunction [81]. In human autopsy studies, multifocal or diffuse necrosis of the cerebral cortex, basal ganglia, and hippocampus was observed in patients who died of hypoglycemia [82]. A dose-response relationship between the occurrence of severe hypoglycemic episodes and risk of dementia development has been reported in a retrospective study involving 16,667 T2DM patients [84]. Although contradicting results have been reported by some studies [85, 86], arguing that tolerance to a hypoglycemic state can be developed in patients exposed to hypoglycemia chronically, the effect of hypoglycemia on some high-risk groups cannot be ignored. For example, it has been shown that impairment of memory functioning is strongly correlated with severe hypoglycemia in T1DM patients [87].

#### 3.2.3. Changes in Insulin and Amyloid Metabolism

The blood glucose level is regulated by insulin, a hormone generated by the beta cells in the pancreas. Traditionally, it was believed that the brain is an insulin independent organ; however, recent studies have suggested otherwise [88]. It has been shown that insulin is actively transported across the blood brain barrier [89] and is also produced locally in the brain [90]. Furthermore, insulin receptors are expressed in the hippocampus and the cortex, indicating its functional role in the brain [91]. In addition, being a regulator of food intake and energy homeostasis [88], insulin also plays a role in memory and learning [92]. Changes in insulin levels and receptor sensitivity could lead to deficits in cognitive function [93]. In AD, impairments of cerebral insulin receptors activation and elevated insulin level in the CSF have been reported [94], indicating the contribution of insulin in cognitive decline and dementia development.

Hyperinsulinemia is a common characteristic of T2DM and has been identified as a risk factor for cognitive dysfunction and dementia progression [95, 96]. It has been suggested that hyperinsulinemia is associated with reduction of amyloid metabolism, due to downregulation of insulin-degrading enzyme (IDE) levels in the brain [97]. IDE is responsible for the degradation of insulin and amyloid-*β* peptide (A*β*). Therefore reduced IDE levels would lead to A*β* accumulation in the brain, contributing to AD and cognitive impairment [98]. Hyperphosphorylation of tau protein is another pathological hallmark of AD. It has been suggested that inhibition of insulin-mediated pathways can lead to hyperphosphorylation of tau and A*β* production, via activation of the glycogen synthase kinase 3 (GSK3) signalling [99, 100].

### 3.3. Inflammation

Inflammation has been implicated in the onset of DM and progression of its complications [95]. It has been suggested that people suffering from DM are under a state of subclinical chronic inflammation [101, 102]. Numerous proinflammatory markers and cytokines, such as C-reactive protein (CRP), tumour necrosis factor- (TNF-) *α*, interleukin- (IL-) 1*β*, and IL-6, have been shown to be upregulated in both T1DM and T2DM [103]. Many of the proinflammatory markers have been associated with cognitive decline and dementia development [104]. Given the fact that many of these inflammatory markers found in DM patients are closely associated with the pathogenesis of AD [105], there is an increasing interest in the link between DM and dementia. Yaffe et al. reported that impaired cognitive functions were observed in DM patients with elevated CRP and IL-6 levels, but not in patients with normal levels of these markers [71].

Inflammation has been suggested to induce cerebral changes via multiple mechanisms. Firstly, it has been shown that chronic inflammation in DM can induce changes in blood brain barrier (BBB) permeability [106]. Increase in BBB permeability has been observed in brain biopsies from AD patients [107]. Moreover, increased BBB permeability can also allow access of toxic substances and metabolites into the brain, leading to cerebral damage [108]. Secondly, neuroinflammation is a well-established factor in the development of cognitive decline, dementia, and other neurodegenerative diseases [109, 110]. It has been demonstrated that inflammatory cytokines can cause activation of glia cells leading to neuronal damage. For example, TNF-*α* has been shown to induce hippocampal dysfunction, via activation of the JNK and the I*κ*B*α* kinase/NF*κ*B signalling pathway [111–113]. Finally, inflammation plays a central role in the development of complications in vasculature, including stroke [114], contributing to cognitive impairment and dementia development as discussed in Section 3.1.

## 4. The Use of TCM in the Treatment of Cognitive Dysfunction and Dementia in DM Patients

Given the fact that DM is now an established risk factor for cognitive dysfunction and dementia, there is an increasing interest in targeting DM for the treatment of cognitive decline and dementia. Several studies suggested that oral antidiabetic drugs such as thiazolidinedione and metformin could offer therapeutic benefits to reduce DM-related cognitive dysfunction in both patients and animal models [17, 18]. A recent study demonstrated that glycemic control using empagliflozin significantly prevented cognitive impairment via attenuation of cerebral oxidative stress and increase in cerebral brain-derived neurotropic factor in a T2DM mouse model [115]. This highlighted the potential of antihyperglycemic agents in the treatment of T2DM-related cognitive dysfunction. However, some clinical studies have demonstrated rather limited and inconsistent benefits of these glucose lowering agents in limiting cognitive decline and dementia development [14–16]. Furthermore, the risks associated with the use of antiglycemic/insulin therapies have raised the concerns regarding the long-term safety and effectiveness of these interventions for the management of DM-induced cognitive dysfunction and dementia [84, 85]. For example, hypoglycemia is commonly observed in T1DM patients with tight glycemic control and in advanced T2DM patients [116, 117]. Although intensive insulin therapy has shown to successfully control glycemia and reduce vascular complications in DM patients, several reports have highlighted possible neuronal damage and cognitive impairment due to the incidence of hypoglycemia associated with the insulin therapy [118].

Traditional Chinese medicine (TCM) has been used to treat DM for over thousands of years. A large number of TCM interventions belonging to several key modalities (such as herbal medicine, acupuncture, and Taichi) have been used for the management of DM and its complications [19] (Table 1). According to traditional Chinese medical system, Chinese practitioners, who often adopt a holistic approach in treating their patients, manage diabetes through integrated care: nourishing and strengthening the body's functions rather than focusing solely on blood glucose control [119, 120]. Despite a lack of scientific validation of the TCM interventions for diabetes, the accumulated evidence has demonstrated some promising results in relieving the symptoms and complications of diabetes [121, 122]. In this section, we will firstly review the current clinical findings of TCMs used for DM-related cognitive dysfunction and then highlight some of the potential mechanisms underlying the TCM effects on the condition.

### 4.1. Clinical Studies

Numerous clinical studies have demonstrated the beneficial effects of TCM interventions on cognitive dysfunction and dementia. Data from a meta-analysis suggest that TCM interventions appear to be a safer and more effective treatment for vascular dementia, based on 31 randomised clinical trials comparing 1605 patients on TCM treatments with 1263 patients on Western medicine or placebo [123]. It has been suggested that sleep apnea hypopnea syndrome (SAHS) patients with T2DM have a higher risk of cognitive decline than the nondiabetic SAHS patients [116]. Interestingly, in a clinical study, a 2-week treatment with DanHong Injection, consisting of extracts of* Salvia miltiorrhiza* and* Carthamus tinctorius* L., significantly improved the Montreal Cognitive Assessment (MoCA) score, especially in the executive function and memory domains, in 86 SAHS patients with T2DM. Although blood glucose levels were not examined in this study, the results indicate that DanHong Injection could improve cognitive function in T2DM patients [124]. Another clinical study in 36 T2DM patients has demonstrated that combined Huang Qi (*Radix Astragali*) and Chuanxiong (*Ligusticum Wallichii*) injections over 30 days significantly reduced blood glucose levels and improved cognitive function, whilst the standard pharmaceutical care with antidiabetic agents in the control group only reduced the blood glucose levels, indicating that the herbal formula may provide therapeutic benefits to DM patients beyond its blood glucose lowering property [125].

In a clinical trial involving 164 diabetic patients with complicated coronary heart disease (CHD), it was shown that while both isosorbide mononitrate (20 mg, twice a day) and FufangDanshenDiwan (consisting of* Salvia miltiorrhiza*,* Panax pseudoginseng* var.* notoginseng*, and* Dryobalanops aromatica* Gaertn.* f.*) (270 mg, once daily) treatments for 16 weeks significantly improved the cardiac ischemia burden (as measured by 24-hour ambulatory ECG monitoring), improvements in insulin sensitivity and lipid metabolism indexes were only observed in patients received FufangDanshenDiwan. Moreover, the herbal formula was also shown to reduce A*β* formation and improve cognitive function in the DM patients [149]. As mentioned in Section 3, increased oxidative stress and inflammation are closely associated with the cognitive dysfunction in DM patients. Li and Yeung have demonstrated that an 8-week treatment with Zhi Nao capsule consisting of extracts of* Codonopsis pilosula*,* Polygonatum sibiricum*,* Ligusticum Wallichii*, and* Acorus tatarinowii* significantly increased serum superoxide dismutase (SOD), reduced CRP level, and limited cognitive decline and dementia development in T2DM patients [150].

In China, it is not an uncommon practice to use integrative strategies, combining TCM and Western medicine interventions, in the treatment of DM and its complications [151]. Numerous studies have assessed the efficacy of the combined therapies to treat cognitive dysfunction in DM patients. Nao Xin Tong, a complex herbal formula (consisting of* Radix Astragali*,* Salvia Miltiorrhizae*,* Angelicae Sinensis*,* Ligusticum Wallichii*,* Paeoniae Rubra*,* Flos Carthami Tinctorii*,* Gummi Olibanum*,* Resina Commiphorae Myrrhae*,* Ramulus Cinnamomi Cassiae*,* Buthus martensi*,* Lumbricus*, and* Hirudo seu Whitmaniae*) has been shown to improve cognitive function in 32 stroke patients with T2DM [152]. In patients who received a combined therapy of alprostadil with Lao Xin Tong, a greater cognitive enhancing effect was observed when compared to the alprostadil only group [153]. In a clinical trial, 48 DM patients with VaD were randomised to receive piracetam or piracetam plus oral ShengmaiDingzhi decoction (consisting of* Pseudostellaria heterophylla* (Miq.) Pax,* Ophiopogon japonicus*,* Schisandra chinensis*,* Wolfiporia extensa*,* Polygala tenuifolia* Willd.,* Acorus tatarinowii*,* Pinellia ternata* (Thunb.) Breit,* Semen Persicae*,* Panax pseudoginseng* var.* notoginseng*, and* Glycyrrhiza uralensis*) over 60 days. The combined therapy group demonstrated greater improvements in the Activities of Daily Living Scale (ADLS) and the Scale of Elderly Cognitive Functions (SECF) when compared to that of the piracetam only group [154]. Several studies have demonstrated that a combined therapy of BushenQuyuYizhi decoction (consisting of* Cistanche deserticola*,* Acorus tatarinowii*, and* Panax pseudoginseng* var.* notoginseng*) with nimodipine produced a significantly greater effect than nimodipine alone on cognitive function [155, 156]. In a more recent study conducted by Zhao et al., 85 patients with DM-mediated vascular cognitive dysfunction were allocated to receive a 6-month treatment of aspirin (100 mg, once daily) or aspirin (100 mg, once daily) plus BushenQuyuYizhi decoction (2.5 g per day, orally), over 6 months. At the completion of the 6-month treatment, cognitive dysfunction was improved in both treatment groups. However, the cognitive improvement was only maintained in the combined therapy group 12 months after the treatment, indicating a potential long-term effect of the herbal intervention, possibly via enhancement of general health and limiting disease progression in the patients.

Electroacupuncture is a form of acupuncture by way of applying a small electric current between pairs of fine needles that are inserted into acupoints selected according to the TCM theory [157]. Numerous studies have shown positive effects of electroacupuncture on cognitive impairment [158]. Several recent studies have also demonstrated that electroacupuncture improved cognitive function and quality of life in diabetic patients [159, 160]. Although the sample sizes were small (ranging from 25 to 32 patients) in these studies, the therapeutic potential of electroacupuncture should not be ignored and further investigations with a rigorous design are warranted.

### 4.2. Mechanisms of TCMs in Treating DM-Mediated Cognitive Dysfunction and Dementia

A large number of* in vitro* and* in vivo* preclinical studies have been conducted to assess the underlying mechanisms of TCM interventions in diabetes-related cognitive dysfunctions. Reduced antioxidative levels and increased ROS generation are closely associated with the pathogenesis of diabetes and its complications. Antioxidant properties of CHMs have been demonstrated in numerous studies [161, 162]. For example, green tea, which is commonly consumed in Eastern and Asian countries, contains of a mixture of plant polyphenols that possess antioxidative and radical-scavenging activities [163]. In obese KK-ay mice, green tea catechins reduced blood glucose levels and insulin resistance via inhibition of the TNF-*α*-induced ROS generation [164]. It has also been shown that green tea catechins markedly suppressed memory regression in SAMP10 mice, a mouse model of brain senescence with cerebral atrophy and cognitive dysfunctions. Daily consumption of green tea catechins significantly reduced brain atrophy and suppressed DNA oxidative damage. These effects were associated with the improvement of plasma antioxidative activity caused by daily green tea consumption [165]. A recent study showed that green tea catechins remarkably ameliorated learning and memory impairments in a diabetic rat model, via the reduction of oxidative stress and nitric oxide modulation. In this study, green tea catechins also significantly reduced the blood glucose levels, indicating that green tea can suppress diabetes-mediated cognitive dysfunction via both hypoglycemic and antioxidative effects [166]. Indeed, numerous other CHMs, such as berberine and ginsenoside, have been shown to reduce diabetes-mediated cognitive decline via reduction of oxidative stress [167, 168].

Tanshinol (TSL), a bioactive component of Danshen (*Salvia miltiorrhiza*), widely used for vascular disease [169], was shown to improve spatial working memory and attenuated vascular dementia in rats, via an increase in acetylcholine levels and reduction of acetylcholinesterase activity [170]. Tanshinone IIa (Tan IIa), another bioactive component of Danshen, was also shown to restore diabetes-induced nerve deficiency [171]. The regulation of the cholinergic neurotransmission in the brain plays a vital role in memory and cognitive function. Similar to TSL, several other herbal constituents, including lycopene, berberine, and curcumin, have been shown to ameliorate diabetes-related cognitive dysfunction, via protection of the cholinergic neurotransmission [167, 172, 173].

In addition, several other mechanisms have been proposed as underlying the effect of CHMs on DM-mediated cognitive dysfunction, such as through reduction of AGEs-mediated neuroinflammation and the downregulation of cerebral amyloid-beta (A*β*). For example, Danshensu, a bioactive component of* Salvia miltiorrhiza*, has been shown to improve learning and memory in diabetic mice via suppression of AGE-mediated neuroinflammation. These effects were independent of blood glucose, insulin, and glycosylated hemoglobin levels, indicating a direct neuroprotective and anti-inflammatory effect of Danshensu [174]. Liuwei Dihuang decoction (LWDHD), a well-established TCM formulation, consisting of six herbs (*Rehmannia glutinosa* Libosch.,* Cornus officinalis* Sieb.,* Dioscorea oppositifolia* L.,* Paeonia ostii*,* Alisma orientale* (G. Samuelsson) Juz., and* Poria cocos* (Schw.) Wolf), has been shown to attenuate neural apoptosis and A*β* deposition in the hippocampus and cerebral cortex in a streptozotocin-induced diabetic rat model [175]. In addition, LWDHD also reduced blood glucose levels, decreased oxidative stress, and suppressed inflammation in hippocampus of the animals [175]. Interestingly, Chen et al. showed that administration of ZiBuPiYin recipe (ZBPYR), a modification of the Zicheng decoction (consisting of 12 herbs:* Panax ginseng* C. A. Meyer,* Dioscorea opposita* Thunb.,* Poria cocos* (Schw.) Wolf,* Paeonia lactiflora* Pall.,* Salvia miltiorrhiza* Bge.,* Dolichos lablab* L.,* Nelumbo nucifera* Gaertn.,* Acorus gramineus* Soland.,* Polygala tenuifolia* Willd.,* Santalum album* Linn.,* Citrus maxima* (Burm.) Merr. cv.* Tomentosa, *and* Glycyrrhiza uralensis* Fisch.), over a 6-week treatment, prevented DM-associated cognitive decline in db/db mice. The observed effect was possibly due to improving dendritic spin density and attenuating brain leptin and insulin signalling pathway injury [176]. Although these findings need to be confirmed in humans, it provides important preclinical data to support the potential benefits of TCM in preventing and slowing the development and progression of DM-associated cognitive dysfunction.

Data from the above studies have clearly indicated that the positive effects of CHMs could be mediated by multiple pathways and mechanisms. Combination therapy underpins the philosophy of CHMs, where patients are generally treated with multiherb formulations. Complex chemical mixtures of CHMs enhance therapeutic efficacy by facilitating synergistic action and/or ameliorating/preventing potential side-effects. The multicomponent and multitarget approach of CHMs makes them ideal therapies for disorders such as DM-mediated cognitive dysfunction and dementia, which have multifactorial/multisystem pathophysiological components.

## 5. Summary and Future Directions

Substantial effort has been invested to understand the effects of diabetes on cognitive decline and dementia in the past decade. Recent studies have identified the risk factors and possible mechanisms underlying the pathogenesis of the DM-mediated cognitive dysfunction. By taking advantage of recent advancements in these processes, it is possible to develop better therapies for DM-related cognitive complications, including AD, vascular dementia, and cognitive decline. Numerous clinical studies have highlighted the potential of TCMs in the treatment of DM-related cognitive decline. Despite the fact that most of these trials have shown positive outcomes, significant methodological issues such as small sample sizes and poor randomization exist in many of these studies. Therefore, more rigorously designed randomized controlled trials are required to further validate these findings. Finally, most of the preclinical studies assessing the effects of CHMs on DM-mediated cognitive dysfunction were performed using single herbs or isolated active ingredients. Given that the traditional use of CHMs is based on complex formulation, more research on the synergic effects of herbal combinations is required for a more comprehensive understanding of the mechanisms underlying their effect on the diseases.

## Figures and Tables

**Table 1:** A summary of key TCM modalities used of the treatment of diabetes.

TCM modalities	Mechanisms of action	References
Herbal medicine		
*Radix Astragali *	Improved hyperglycemia status, insulin sensitivity, and glucose uptake	[126]
*Salvia Miltiorrhiza *	Reduce oxidative stress and improved insulin sensitivity index	[127]
	Reduced serum oxLDL and sVCAM-1	[128]
*Panax ginseng *	Reduced blood glucose and insulin level and inhibited angiogenesis	[129]
	Reduced blood glucose level and improved glucose tolerance	[130]
*Semen Coicis *	Hypoglycemic effect, no effect on HbA1c level	[131]
*Dioscoreae rhizoma *	Hypoglycemic effect and improved insulin sensitivity	[132]
*Ganoderma lucidum *	Reduced serum glucose level and suppressed hepatic PEPCK gene level	[133]
*Cinnamomi Cassiae *	Increased insulin sensitivity, reduced lipids level, and hypoglycemic effect	[134]
	Antioxidative effect and reduced blood glucose level	[135]
*Radix Platycodi *	Increased insulin sensitivity and GLUT4 translocation	[136]
*Ligusticum chuanxiong *	Reduced blood glucose level, improved renal function, and decreased VEGF level	[137]
*Gynostemma pentaphyllum *	Increase SOD and GSH-px activities and improved glycemic control	[138]
*Potentilla chinensis *	Antioxidative and antihyperglycemic effects	[139]

Acupuncture		
Acupuncture to 6 points (Zhongwan, Tianshu, Qihai, Ganshu, Pishu, and ShenShu)	Reduced blood glucose level, no effect on body weight	[140]
Electroacupuncture at Zusanli (ST-36) and Zhongwan (CV-12)	Reduced blood glucose level via stimulation of the cholinergic nerves	[141]
Acupuncture at points GB34 and GB39	Decreased ischemic in brain and neuronal protective effect	[142]
Electroacupuncture at ear points and to body points	Reduced glucose level and increased serum insulin and c-peptide levels	[143]

Taichi		
1-hour session twice a week for 12 weeks	Improved glucose control, balance, and neuropathic symptoms	[144]
1.5-hour session three times a week for 12 weeks	Improved health-related quality of life such as physical functioning and body pain	[145]
19 Taichi movements, twice a week for 6 months	Declined fasting glucose and HbA1c level, better quality of life in mental health	[146]
1 hour per day, 5 days a week for 14 weeks	Improved glycemic control and lowered serum TG level	[147]
12 weeks of Taichi exercise programme	Decreased HbA1c level with increased Th1 reaction and blood IL-12 level	[148]
